# Glutamic-Alanine Rich Glycoprotein from *Undaria pinnatifida*: A Promising Natural Anti-Inflammatory Agent

**DOI:** 10.3390/md22090383

**Published:** 2024-08-26

**Authors:** Md Saifur Rahman, Md Badrul Alam, Marufa Naznin, Mst Hur Madina, S. M. Rafiquzzaman

**Affiliations:** 1 Institution of Nutrition and Functional Foods, Faculty Agricultural and Food Sciences, Laval University, Laval, QC G1V 0A6, Canada; mosammad-hur.madina.1@ulaval.ca; 2 Inner Beauty/Antiaging Center, Food and Bio-Industry Research Institute, Kyungpook National University, Daegu 41566, Republic of Korea; mbalam@knu.ac.kr; 3 Department of Chemistry, Kyungpook National University, Daegu 41566, Republic of Korea; naznin@knu.ac.kr; 4 Department of Fisheries Biology and Aquatic Environment, Bangabandhu Sheikh Mujibur Rahman Agricultural University, Gazipur 1706, Bangladesh; rafiquzzaman@bsmrau.edu.bd

**Keywords:** anti-inflammation, glycoprotein, NF-κB, *Undaria pinnatifida*, MAPK, NSAID

## Abstract

This study aimed to assess the anti-inflammatory properties of a bioactive glutamic-alanine rich glycoprotein (GP) derived from *Undaria pinnatifida* on both LPS-stimulated RAW264.7 cells, peritoneal macrophages, and mouse models of carrageenan- and xylene-induced inflammation, investigating the underlying molecular mechanisms. In both in-vitro and in-vivo settings, GP was found to reduce the expression of inducible nitric oxide synthase (iNOS) and cyclooxygenase-2 (COX-2) while also inhibiting the production of nitric oxide (NO) and prostaglandin E_2_ (PGE_2_) in response to lipopolysaccharide (LPS) stimulation. GP treatment significantly impeded the nuclear translocation of the nuclear factor kappa-light-chain-enhancer of activated B cells (NF-κB) pathway by blocking the phosphorylation of IKKα and IκBα, leading to a reduction in proinflammatory cytokines such as tumor necrosis factor-α (TNF-α), interleukin-1β (IL-1β), and interleukin-6 (IL-6). Additionally, GP effectively inhibited the activation of mitogen-activated protein kinases (MAPKs), with specific inhibitors of p38 and extra-cellular signal regulated kinase (ERK) enhancing GP’s anti-inflammatory efficacy. Notably, GP administration at 10 mg/kg/day (p.o.) markedly reduced carrageenan-induced paw inflammation and xylene-induced ear edema by preventing the infiltration of inflammatory cells into targeted tissues. GP treatment also downregulated key inflammatory markers, including iNOS, COX-2, IκBα, and NF-κB, by suppressing the phosphorylation of p38 and ERK, thereby improving the inflammatory index in both carrageenan- and xylene-induced mouse models. These findings suggest that marine resources, particularly seaweeds like *U. pinnatifida*, could serve as valuable sources of natural anti-inflammatory proteins for the effective treatment of inflammation and related conditions.

## 1. Introduction

Inflammation is a complex process involving multiple steps. However, it remains a pivotal protective immune system reaction that promotes tissue injury by migrating immune cells such as monocytes. Macrophages represent the first (M1 type, classically activated) and second (M2 type, alternatively activated) immune response in eclectic and diverse organisms, including mammals [1]. Upon encountering pathogens or inflammatory signals, macrophages differentiate into the M1 phenotype under the influence of interferon-gamma (IFN-γ) and LPS. These M1 macrophages are characterized by their pro-inflammatory properties, producing high levels of pro-inflammatory cytokines such as TNF-α, IL-1β, and IL-6, as well as reactive nitrogen and oxygen species like NO and superoxide anions. Their primary role is to orchestrate the inflammatory response by phagocytosing pathogens, presenting antigens to T cells, and secreting mediators that recruit and activate other immune cells. This activity is critical for initiating and sustaining the inflammatory response, ultimately aiming to clear infections and trigger tissue repair mechanisms. However, prolonged or excessive activation of M1 macrophages can contribute to chronic inflammation and tissue damage, underlying many inflammatory diseases, such as rheumatoid arthritis and atherosclerosis [2,3]. In addition to their immediate defense against infections, macrophages have recently been shown to upregulate two pivotal factors in epigenetic signaling and chromatin structure and organization: reactive nitrogen species (RNS) and reactive oxygen species (ROS), which are paramount to each other in signaling cell regulatory factors towards a possible path to chronic disease. Research indicates that macrophage polarization and activation are tightly regulated by epigenetic mechanisms, where RNS and ROS play critical roles. These species can induce modifications in histone proteins and DNA, thereby altering chromatin structure and influencing gene expression. For instance, a study highlighted that macrophages exposed to different stimuli produce varying levels of RNS and ROS, which, in turn, modulate the activity of key epigenetic enzymes such as histone deacetylases (HDACs) and Jumonji domain-containing protein 3 (Jmjd3). These enzymes are crucial in macrophage differentiation and activation, impacting the overall inflammatory response and potentially leading to chronic conditions if dysregulated [4]. Additionally, another study on HIV-infected macrophages revealed that RNS and ROS can significantly affect the epigenetic landscape, promoting a unique transcriptional profile that maintains viral persistence and contributes to chronic disease states [5]. Chronic inflammation can progress to a host of major diseases, including arthritis, cancer, and diabetes, all of which highlight the need for therapeutic interventions to address dysfunctional inflammatory pathways. In the innate immune network, pattern recognition receptors (PRRs) play a major role in recognizing external pathogens and their products, with a prominent role in the Toll-like receptor (TLR) family [6]. TLR-4 can respond to various stimuli, including lipopolysaccharides, viral nucleic acids, alarmins (from dead or dying cells), and microparticles made from snack food crumbs [7,8,9,10,11]. Upon binding to these foreign structures, TLR-4 sends signals through different sets of signaling proteins such as MyD88, which later engages NF-κB and triggers the production of inflammatory cytokines. The reason for targeting specific pathways, such as shutting down the activity of NF-κB, is inflammation and its effects [6,12,13].

Carrageenan (CA)-induced inflammation and xylene-induced ear edema in mice are generally used as reproducible and reliable exploratory models of inflammation, and are widely used in drug discovery. This model is well established as it encompasses all stages of inflammation. In the early phase (first three hours), histamine, nitric oxide, and other mediators are secreted by white blood and other cells. In the late phase (second three hours), stressed cells release long-lasting cytokines [14,15].

Synthetic nonsteroidal anti-inflammatory drugs (NSAIDs) are widely prescribed for the treatment of inflammatory diseases. Nevertheless, prolonged use of these artificial substances, such as NSAIDs, can lead to significant adverse reactions, including gastrointestinal disorders [16]. Therefore, there is a significant interest in the development of new natural bioactive peptides and protein drugs to address inflammation. In the search for natural drug sources, many scientists have focused on marine resources for natural anti-inflammatory components, particularly edible seaweeds [17]. Research on marine algae has identified 86 inhibitors, some of which are commercially available [18]. Like *Ascophyllum nodosum*, seaweed extracts deliver health benefits through various bioactive components. Numerous studies have indicated that seaweeds and their organic extracts contain numerous beneficial bioactive components [19]. *Undaria pinnatifida*, a brown alga from East Asia, is a popular food source [20]. Zhang et al. (2014) found that bioactive compounds such as fucoidan, fucoxanthin, phlorotannin, PUFA, and peptides from *U. pinnatifida* have antimicrobial, antidiabetic, antioxidant, anticancer, antiviral, and anti-inflammatory properties [21]. Although *U. pinnatifida* glycoprotein exhibits a wide range of biological activities, its pharmacological properties have not yet been thoroughly explored [22,23,24]. We purified GP from *U. pinnatifida* to study its biofunctional effects on *Lactobacillus plantarum*, including antioxidant, antidiabetic, prebiotic, and DNA-protective [25,26,27]. The glycoprotein purified from *U. pinnatifida* exhibits well-defined chemical composition and structural characteristics. Comprising 42.53% carbohydrates and 57.47% proteins, GP was linked via O-glycosylation, as indicated by its amino acid profile, FT-IR spectrum, and enzymatic glycosylation analysis. SDS-PAGE analysis revealed that GP migrated as a single band with an approximate molecular weight of 10 kDa, which was confirmed by Schiff’s reagent staining. Detailed analysis of its amino acid and monosaccharide profile shows a rich composition of glutamic acid, alanine, aspartic acid, and various monosaccharides, underscoring its potential as a bioactive compound [25,26,27].

However, there is only preliminary information regarding the anti-inflammatory properties of *U. pinnatifida* glycoprotein. This study details the anti-inflammatory effects of *U. pinnatifida* glycoprotein on LPS-activated macrophages, CA, and xylene-induced inflammatory mice by regulating MAPKS and the nuclear factor kappa-light-chain-enhancer of activated B cells (NF-κB) signaling axis.

## 2. Results

### 2.1. Cell Viability and Inhibitory Effect of GP on NO and PEG_2_ in LPS-Stimulated RAW 264.7 and Peritoneal Cells

**Figure 1:**
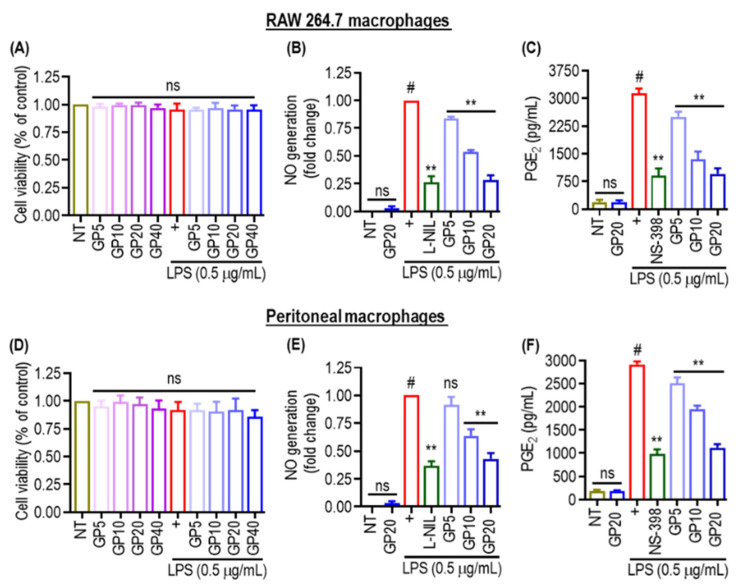
Cell viability and inhibitory effect of GP on NO and PEG_2_ in LPS-stimulated RAW 264.7 and peritoneal cells. (**A**,**D**) Viability of RAW 264.7 macrophages and peritoneal macrophages, respectively, after exposure to 0.5 μg/mL LPS. Viability was expressed as a percentage of the non-treated control (NT) and non-significant (ns) changes in cell viability, indicating no cytotoxic effects. (**B**,**E**) NO generation in RAW 264.7 and peritoneal macrophages, respectively. L-NIL/NS-398 was used as a positive control. (**C**,**F**) Production of PGE_2_ in RAW 264.7 and peritoneal macrophages, respectively. Statistical significance is indicated as follows: ns (not significant); (#) indicates *p* < 0.05 vs. NT. (**) indicates *p* < 0.05 vs. LPS alone. The data are shown as the mean ± SD of three independent experiments.

The effect of GP treatment on two types of macrophages, RAW 264.7 and peritoneal (Figure 1), was studied. These cells are commonly used to examine inflammation and immunological reactions. The cell viability results shown in Figure 1A,D indicate that the treatments did not induce cytotoxicity. NO is a crucial signaling molecule involved in the immune response. Elevated NO production is commonly associated with inflammatory reactions [28]. Figure 1B,E demonstrates that the administration of L-NIL (a selective iNOS inhibitor) led to a considerable decrease in NO production in both cell types compared to the control group treated solely with LPS. GP exhibited a substantial decrease in NO levels in RAW 264.7, and peritoneal macrophages at concentrations of 5, 10 and 20 μg/mL.

Remarkably, 20 µg/mL GP showed equivalent inhibition of NO production in RAW 264.7 cells as L-NIL. Furthermore, In Figure 1C,F, PGE_2_ is a substance that causes inflammation. GP significantly decreases the synthesis of PGE_2_ in RAW 264.7 macrophage cells in a concentration-dependent manner, and surprisingly, the highest concentration of GP (20 μg/mL) showed an almost similar effect on PGE_2_ inhibition as compared to NS-398 (a selective COX-2 inhibitor) in both macrophage cells. In summary, Figure 1 shows that GP consistently suppressed the production of NO and PGE_2_ without causing any cellular toxic effects.

### 2.2. GP Reduces LPS-Induced iNOS and COX-2 in Macrophages

**Figure 2:**
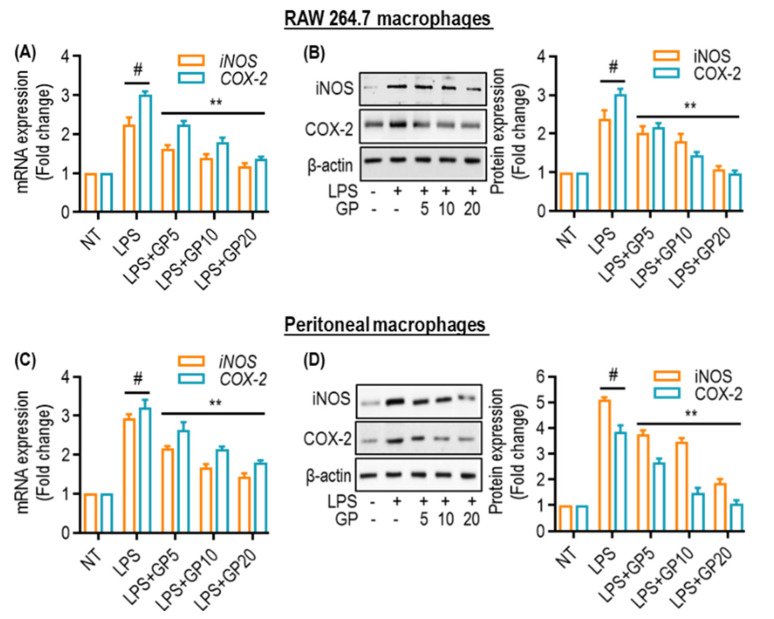
Effects of GP on LPS-induced iNOS and COX-2 in macrophages. (**A**,**C**) Quantitative PCR analysis of iNOS and COX-2 mRNA levels in GP treated (5, 10, and 20 µg/mL) RAW 264.7 and peritoneal macrophages. (**B**,**D**) Quantification of iNOS and COX-2 protein expression levels in RAW 264.7 and peritoneal macrophages. The fold change is normalized to NT using β-actin as a loading control. (#) indicates *p* < 0.05 vs. NT; (**) indicates *p* < 0.05 vs. LPS alone. The data are shown as the mean ± SD of three independent experiments.

We examined the effects of GP on iNOS and COX-2 in RAW 264.7 and peritoneal macrophages. LPS stimulates macrophages to enhance inflammation and generate iNOS and COX-2. RAW 264.7 cells and peritoneal macrophages were exposed to LPS, and there was a noticeable increase in iNOS and COX-2 mRNA levels and protein expression (Figure 2). GP effectively reduced the release of iNOS and COX-2 mRNA in a concentration-dependent manner (Figure 2A,C). This effect was observed at concentrations of 5, 10, and 20 µg/mL. GP also decreased the protein expression of iNOS, and COX-2 induced by LPS (Figure 2B,D). Therefore, GP may be highly effective at reducing inflammation.

### 2.3. Cytokine Expression and Secretion in Macrophage Cultures

**Figure 3:**
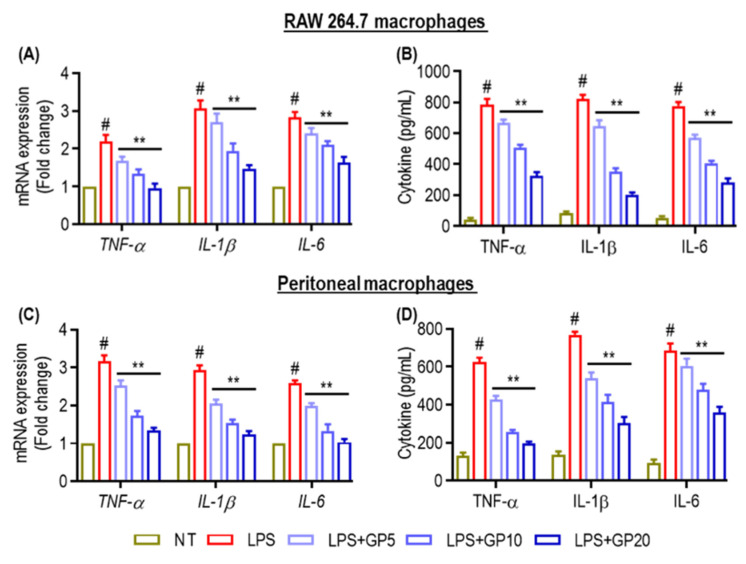
Cytokine expression and secretion in macrophage cultures. (**A**,**C**) Quantitative analysis of mRNA expression levels of TNF-α, IL-1β, and IL-6 in RAW 264.7 and peritoneal macrophages. Treatments included non-treatment (NT), LPS alone, or co-treatment with GP at 5, 10, and 20 μg/mL. (**B**,**D**) TNF-α, IL-1β, and IL-6 levels were measured in pg/mL in RAW 264.7 and peritoneal macrophages. (#) indicates *p* < 0.05 vs. NT; (**) indicates *p* < 0.05 vs. LPS alone. The data are shown as the mean ± SD of three independent experiments.

The effects of LPS and its combination with various concentrations of GP on cytokine production were assessed in RAW 264.7 macrophage (Figure 3A,B) and peritoneal macrophages cultures (Figure 3C,D). Treatment with LPS significantly upregulated the mRNA expression and secretion of TNF-α, IL-1β, and IL-6 in both cell types compared with non-treated (NT) controls. Co-treatment with GP (5, 10, and 20 μg/mL) and LPS resulted in a concentration-dependent decrease in both mRNA expression and cytokine secretion (Figure 3). This trend was consistent across all three cytokines and for both RAW 264.7 cells and peritoneal macrophages, indicating that GP may exert a suppressive effect on LPS-induced cytokine production. Statistical analysis supported these observations, identifying significant differences between the LPS-only treated groups and those receiving LPS and GP co-treatment.

### 2.4. Effects of GP on NF-κB Activation Pathway in LPS-Stimulated Macrophages

**Figure 4:**
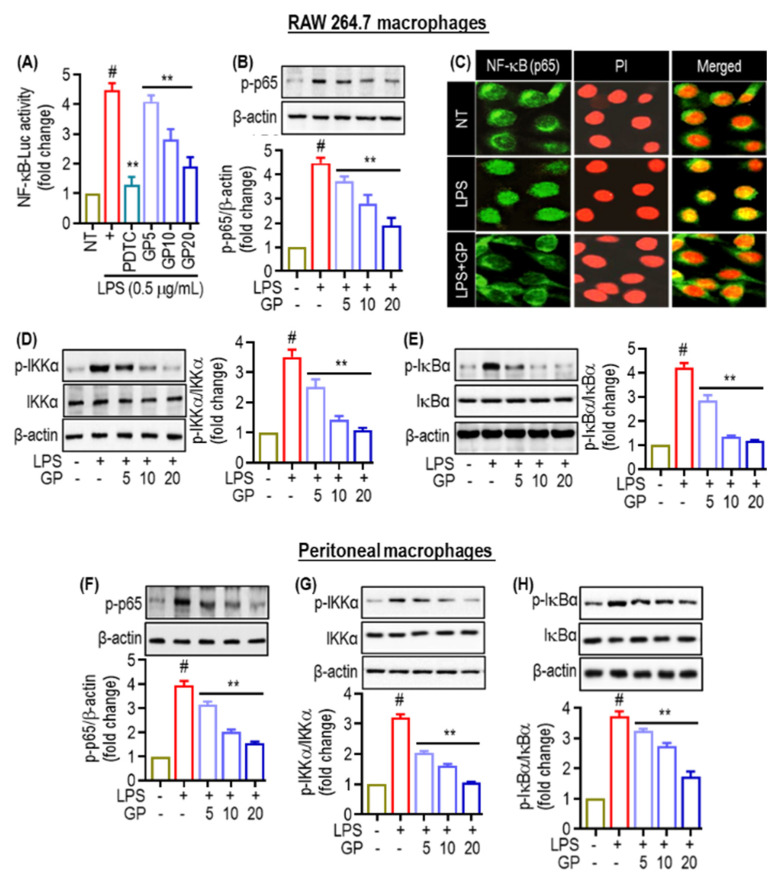
Effects of GP on NF-κB activation pathway in LPS-stimulated macrophages. (**A**) RAW 264.7 macrophage NF-κB luciferase reporter assay evaluating GP pre-treatment. To reduce NF-κB activity, cells were non-treated (NT), treated with LPS (0.5 μg/mL), or pre-treated with GP at 5, 10, or 20 μg/mL before LPS stimulation. (**B**,**F**) Western blot of RAW264.7 and peritoneal cell phosphorylated p65 (p-p65). (**C**) Immunofluorescence staining displays NF-κB p65 subunit (green) and nuclei (red, PI-stained) in RAW 264.7 cells. (**D**,**G**) Western blot analysis shows the expression of phosphorylated IKKα (p-IKKα) in RAW264.7 and peritoneal macrophages after GP administration. (**E**,**H**) In RAW264.7 and peritoneal macrophages treated with GP before LPS exposure, Western blot analysis showes expression of phosphorylated IκBα (p-IκBα). (#) indicates *p* < 0.05 vs. NT; (**) indicates *p* < 0.05 vs. LPS alone. The data are shown as the mean ± SD of three independent experiments.

Pro-inflammatory mediators are produced by activated RAW264.7 and peritoneal macrophages, which are controlled by the transcription factor NF-κB. We evaluated the role of GP in suppressing the NF-κB signaling pathway. LPS stimulation increased p65 phosphorylation in RAW 264.7 cells (Figure 4A). However, pre-treatment with pyrrolidine dithiocarbamate (PDTC), a known anti-inflammatory agent, and GP lowers p-p65, known as phosphorylated p65, a modified form of the p65 subunit of the NF-κB, levels in the nucleus. As shown in Figure 4B,F, the distribution of p65 in the cytoplasm was observed by immunofluorescence labeling before treatment. After LPS treatment, there was a noticeable shift in p65 from the cytoplasm to the nucleus within 1 h, as indicated by the intense nuclear p65 staining. After LPS treatment, there was an increase in p65 nuclear translocation. However, GP therapy decreased p65 nuclear translocation (Figure 4C). To investigate the effect of GP on the breakdown of IκBα, a Western blot analysis was performed to determine cytoplasmic levels (E and H). After 3 h of LPS treatment, there was a significant decrease in IκBα degradation in the cells, as shown in Figure 4E,H. The phosphorylation of IκBα by IKKα/β is crucial for NF-κB activation. IKK/β phosphorylates IκBα, leading to its ubiquitination. We investigated the effect of GP on the inactivation of NF-κB in RAW 264.7 by examining its effect on IKKα/β activation (Figure 4D,G). Thus, LPS stimulation increased IKKα/β phosphorylation, whereas GP pre-treatment significantly lowered IKKα/β phosphorylation (Figure 4D,G).

### 2.5. GP Attenuates LPS-Induced Activation of MAPK Pathways

**Figure 5:**
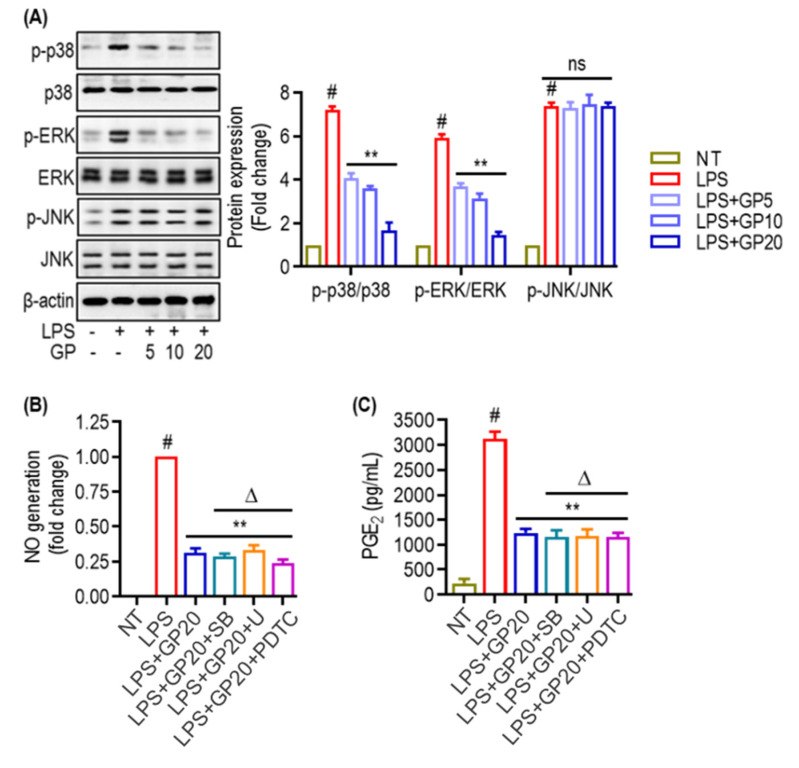
GP attenuates LPS-induced activation of MAPK pathways. (**A**) Representative Western blots and densitometry analysis of phosphorylated and total p38 (p-p38/p38), ERK (p-ERK/ERK), and JNK (p-JNK/JNK) in cells treated with LPS and varying concentrations of GP (5, 10, 20 µg/mL). β-actin serves as a loading control. (**B**) NO production was quantified by Griess assay in cells pre-treated with GP (20 µg/mL) or PDTC before LPS stimulation. NO levels are expressed as fold change over NT (non-treated) control. (**C**) ELISA measured PGE_2_ concentrations in cell culture supernatants after treatment with LPS, GP, and PDTC. Δ *p* < 0.05 compared to LPS + GP20 treatment. ‘ns’ denotes not significant. (#) indicates *p* < 0.05 vs. NT; (**) indicates *p* < 0.05 vs. LPS alone. The data are shown as the mean ± SD of three in-dependent experiments.

The MAPK signaling pathways are essential in regulating the synthesis and release of inflammatory mediators by macrophages during inflammation. To explore how GP influences LPS-induced MAPK activation, we analyzed the phosphorylation status of p38, ERK, and JNK using Western blotting. Additionally, we assessed the production of NO and PGE_2_ in LPS-stimulated RAW 264.7 cells, employing selective inhibitors for p38 (SB239063) and ERK (U0126). As depicted in Figure 5A, LPS treatment significantly increased the phosphorylation of p38, ERK, and JNK, indicating activation of these signaling pathways. GP administration at concentrations of 5, 10, and 20 µg/mL led to a concentration-dependent decrease in the phosphorylation of p38 and ERK. Notably, p38 phosphorylation was significantly reduced at all GP concentrations compared to the LPS-only group, while ERK phosphorylation also decreased in response to GP treatment at these levels. However, GP did not significantly affect JNK phosphorylation, suggesting a selective impact of GP on specific MAPK pathway components.

To determine whether the observed reduction in MAPK signaling was associated with decreased inflammatory responses, we measured NO production using the Griess assay (Figure 5B) and PGE_2_ levels in the cell culture supernatants using ELISA (Figure 5C). LPS exposure led to a significant increase in NO and PGE_2_ production (Figure 5B,C). GP treatment significantly reduced both NO and PGE_2_ levels, with the highest concentration (20 µg/mL) showing the greatest inhibitory effect. Remarkably, the use of MAPK inhibitors not only prevented LPS-induced NO and PGE_2_ production in RAW 264.7 cells but also enhanced the anti-inflammatory effects of GP. These results suggest that the anti-inflammatory effects of GP may be mediated by inhibiting MAPK phosphorylation in activated macrophages, potentially leading to the downregulation of NF-κB signaling pathways. This conclusion is further supported by the observation that PDTC, an NF-κB inhibitor, resulted in an even more pronounced reduction in NO production, indicating that GP’s anti-inflammatory effects may be exerted through the NF-κB pathway.

### 2.6. Effects in Models of Paw Inflammation and Ear Edema

**Figure 6:**
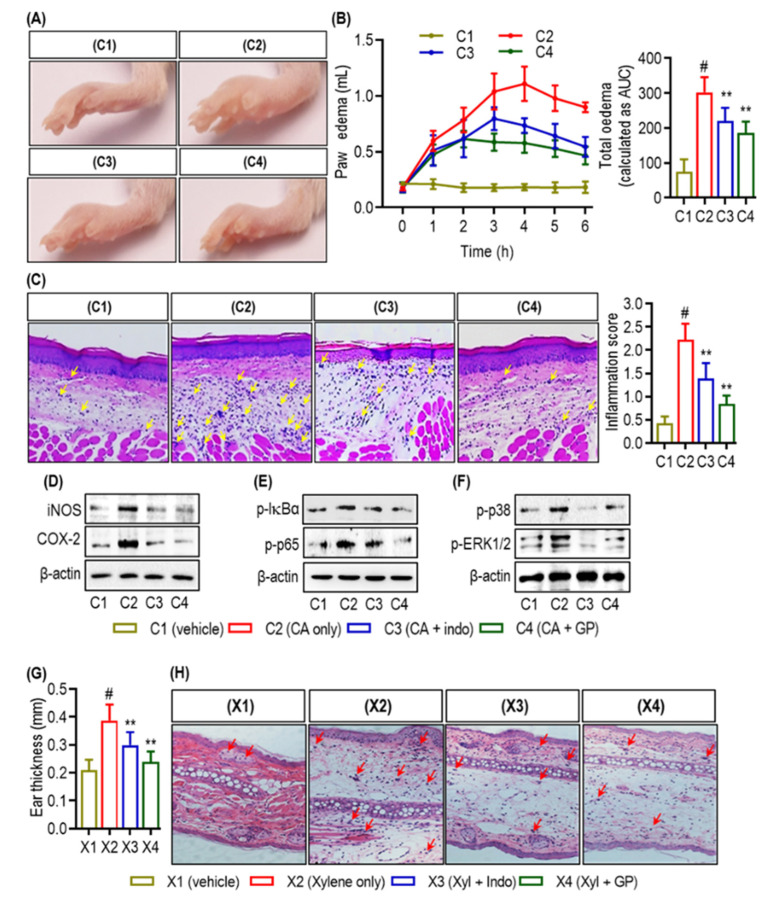
Effects in models of paw inflammation and ear edema. (**A**–**C**) The experimental six mice/group. The control, C1, received dH_2_O; C2, carrageenan-induced inflammation; C3, indomethacin; and C4, GP (10 mg/kg/day, p.o.) for 6 days. (**A**) Representative paw images from each group indicate carrageenan-induced inflammation. (**B**) Paw edema development over 6 h post-carrageenan injection (left *y*-axis) and overall edema volume (right *y*-axis) for each group. (**C**) Hematoxylin–eosin-stained paw tissue sections with yellow arrows suggest inflammatory cell infiltration. Right: quantified inflammatory score. (**D**–**F**) Western blot analyses show iNOS and COX-2 expression, NF-κB pathway phosphorylation (p-IκBα and p-p65), and MAPK pathway phosphorylation (p-p38 and p-ERK1/2) among groups. (**G**,**H**) The experimental mice groups had six mice each. X1 received dH_2_O as the control, X2 received xylene (xyl)-induced ear edema, X3 received indomethacin (indo), and X4 received GP (10 mg/kg/day, p.o.) for 6 days. In the xylene-induced ear edema model, ear tissue thickness demonstrates edema severity for each group. Edema and inflammatory cell infiltration are visible in stained ear tissue (red arrows). A hash symbol (#) indicates a significant difference from C1 or X1, whereas an asterisk (**) indicates a significant difference from C2 or X2 (*p* < 0.05).

Figure 6 illustrates the effects of various treatments on carrageenan-induced inflammation and xylene-induced ear edema. The visual appearance of carrageenan-induced inflammation in the paws of experimental animals is shown for all four groups (Figure 6A,B). As described in Figure 6B, Group C2 was subjected to CA-induced paw edema without any treatment, presenting noticeable edema compared to C1. Group C4 was treated with GP (10 mg/kg/day) and exhibited reduced inflammation, which was better than C3, the indomethacin-treated group.

Histological examination of the paw sections in Figure 6C revealed significant infiltration of inflammatory cells (marked with red arrows) in C2 compared with C1, and remarkably, was markedly reduced in C3 and C4. The inflammation score, shown on the right, quantitatively supported these observations (Figure 6C). Western blot analysis presented in Figure 6D–F shows the expression levels of the inflammatory markers iNOS and COX-2 and the phosphorylation status of NF-κB and MAPK signaling molecules. The C2 group displayed high expression levels of these markers compared to C1, indicative of inflammation, whereas C3 and C4 showed decreased expression, further corroborating the anti-inflammatory effects of the GP treatment.

Figure 6G quantifies the ear thickness in the xylene-induced ear edema model. X2 was subjected to xylene-induced edema and showed the most significant ear thickness compared with X1. Treatment with GP in X4 markedly reduced ear thickness. Interestingly, group X4 showed superior ear edema reduction compared to X3. Histochemical analysis revealed that group X2 exhibits pronounced edema with increased inflammatory cell infiltration (indicated by red arrows). In contrast, sections X3 and X4 displayed reduced edema and inflammation (Figure 6H). Overall, the results suggest that GP treatment effectively reduced carrageenan-induced inflammation and xylene-induced ear edema, with prolonged treatment providing more substantial anti-inflammatory effects.

## 3. Discussion

Inflammatory disorders significantly impact quality of life, with drugs like antibiotics, glucocorticoids, and NSAIDs offering treatment but often causing substantial adverse effects over prolonged use [16]. The potential of bioactive proteins in treating health conditions, including antibiotic and immune system regulation, has drawn scientific and medical interest. This trend has led researchers to study the effects of peptides and protein complexes on the immune response, particularly focusing on natural peptide and protein medicines for treating various ailments. GP from the edible alga *U. pinnatifida* demonstrates significant antimicrobial and antioxidant characteristics, making it a promising candidate for further biological research [25,26,27]. Pharmacological studies have shown that PGE_2_ synthesis and release worsen inflammation. COX-2 is a significant element, and its expression increases PGE_2_ levels, which induces and exacerbates inflammation [29]. Inhibition of PGE_2_ with selective therapies such as NSAIDs reduces inflammation [30]. Our experiments verified this idea by showing that GP-treated cells had lower PGE_2_ and COX-2 protein expression levels than the control cells [28]. The GP treatment stopped this process.

**Figure 7:**
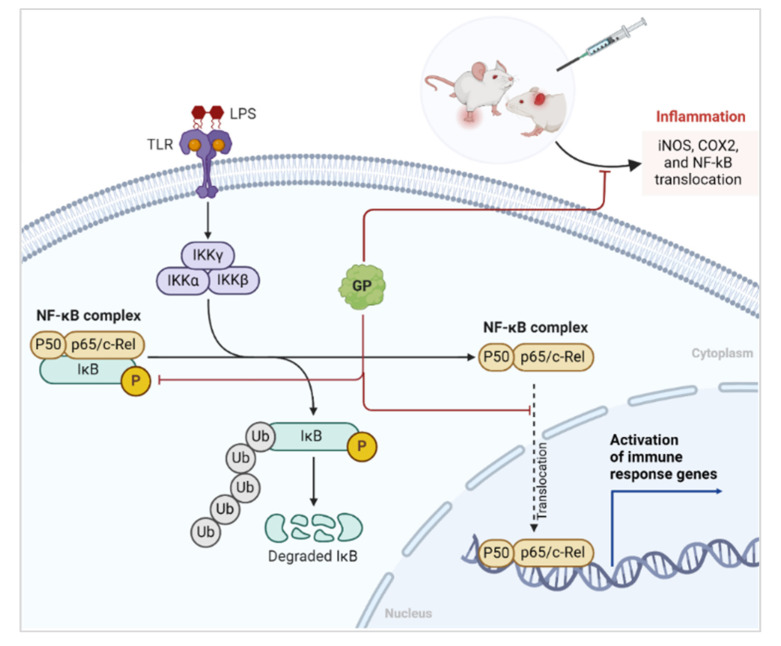
Mechanism of GP in inhibiting NF-κB-mediated inflammation.

The transcription factor NF-κB promotes inflammation by activating inflammation-associated genes [31,32]. NF-κB signal transduction comprises two pathways: canonical and non-canonical. The canonical pathway is associated with inflammation, whereas the non-canonical pathway plays a role in the maturation and differentiation of secondary lymphoid organogenesis and immune cells [33]. NF-κB focuses primarily on COX-2, which is regulated by various inflammatory stimuli and mediators. This leads to upregulation of COX-2 expression and subsequent inflammation. Research indicates that inflammatory illnesses are caused by overexpression of TNF-α, IL-1β, and IL-6. Simultaneously, IL-1β stimulates extracellular cells and activates NF-κB to enhance the inflammatory response [34]. GP administration significantly reduced the levels of TNF-α, IL-1β, and IL-6. It lowered NF-κB expression and translocation into the nucleus, indicating its anti-inflammatory activity and its potential mediation by down-regulation of pro-inflammatory cytokines and NF-κB in RAW264.7 and peritoneal macrophages (Figure 6 and Figure 7).

Inflammation is intricately linked to several signaling pathways, with the transcriptional regulator NF-κB playing a crucial role in the development of various inflammatory diseases. NF-κB modulates the production of numerous cytokines and mediators [35]. NO, produced primarily through the activity of iNOS in activated macrophages, is a critical mediator in the immune response, contributing to the defense against pathogens but also playing a role in the pathogenesis of inflammatory conditions when produced in excess [36]. The iNOS enzyme is typically upregulated in response to inflammatory stimuli, leading to elevated NO levels, which can cause tissue damage if not properly regulated. The MAPK signaling pathways, including p38, ERK, and JNK, are integral to the cellular response to inflammation as they regulate the expression of iNOS and other pro-inflammatory mediators [37]. It has been shown that MAPKs modulate the NF-κB pathway, and inhibition of the MAPK pathway can suppress NF-κB activation [38,39]. We hypothesize that the anti-inflammatory effects of GP are likely mediated through the inhibition of both MAPK and NF-κB signaling pathways, with the suppression of NF-κB being partially due to MAPK inhibition. In this study, the inhibition of the MAPK pathways by the GP was demonstrated to reduce iNOS expression and NO production, thereby attenuating the inflammatory response. This suggests that targeting MAPK pathways could be a potential therapeutic strategy to control iNOS-mediated NO production and mitigate inflammation. Our findings align with previous research indicating that the MAPK/iNOS/NO axis is crucial in the development and maintenance of inflammatory states, and interventions that modulate this pathway may offer significant benefits in treating inflammatory diseases [40,41].

Two commonly used models for studying inflammation are xylene-induced mouse ear edema and carrageenan-induced rat paw edema. These models have been instrumental in researching new anti-inflammatory drugs [34,42,43]. Several Ganluyin (GLY) herbs have been found to effectively reduce edema in mouse ears and feet, demonstrating their anti-inflammatory properties. Reducing edema is a well-established indicator of decreased inflammation, as edema results from increased vascular permeability and fluid accumulation, which are hallmark features of the inflammatory response. By decreasing edema, the treatment demonstrated its ability to stabilize blood vessels and limit fluid leakage, thereby reducing inflammation. This modulation involves several mechanisms: inhibition of pro-inflammatory cytokines (e.g., TNF-α, IL-1β, and IL-6), stabilization of the vascular endothelium, and reduction in immune cell infiltration to the site of injury. Therefore, the observed reduction in paw edema and ear swelling in mice treated with GP highlights its significant anti-inflammatory effects [44,45].

The molecular mechanism (Figure 7) through which GP exerts its anti-inflammatory effects was studied. The diagram highlights the involvement of the NF-κB signaling pathway, a crucial regulator of inflammatory responses. Under inflammatory conditions induced by LPSs, Toll-like receptors (TLRs) activate the IKK complex (IKKα, IKKβ, and IKKγ), leading to the phosphorylation and subsequent ubiquitination of IκB. The degradation of IκB releases the NF-κB complex (p50 and p65/c-Rel), allowing it to translocate into the nucleus and activate the genes responsible for inflammation, such as iNOS and COX-2.

Figure 7 shows that GP interferes with this pathway by inhibiting the phosphorylation and degradation of IκB. This inhibition prevents translocation of the NF-κB complex into the nucleus, thereby reducing the expression of inflammatory genes. Consequently, the administration of GP resulted in decreased levels of pro-inflammatory mediators, such as COX-2 and PGE_2_, as supported by the experimental data showing lower protein expression in GP-treated cells than in controls.

This study demonstrates the therapeutic potential of naturally generated proteins, such as GP. This advances inflammation research and opens up possibilities for the development of anti-inflammatory medications. The intricate interactions between various cellular and molecular pathways in inflammation highlight the complex sensitivity of the body to damaging stimuli, and the potential of targeted therapies to mitigate them.

The complex interactions between these cellular and molecular pathways make GP a promising treatment for inflammatory and oxidative damages. Understanding the structure of GP is a fascinating topic for future research. This study has several limitations. Most notably, the lack of purified materials prevented the use of different doses in an in vivo concentration-dependent experiment. This limitation makes it more difficult to fully comprehend the dose–response connection, which is essential for therapeutic use. Furthermore, there is still a large gap in the comprehensive investigation of glycoprotein structural types and the associated structure–activity connections, even though much research on the protein has focused on structural and biological studies. Further research on the structural and functional elements of glycoproteins is necessary to discover the critical active functional groups that can guide chemical changes and the development of novel medications.

More thorough pharmacokinetic and pharmacodynamic studies are urgently needed, although pharmacokinetic studies have demonstrated the possible pharmacological effects of bioactive compounds. This would make it easier to understand the mechanisms underlying the in vivo anti-inflammatory effects of glycoproteins in vivo. To completely elucidate the therapeutic potential of glycoproteins, future research should overcome these constraints by obtaining sufficient pure samples for various dosage tests and combining thorough structural investigations with kinetic and dynamic evaluations.

## 4. Materials and Methods

### 4.1. Reagents and Chemicals

All reagents and cell culture media were obtained from Sigma-Aldrich (St. Louis, MO, USA). All PCR primers were provided by Bioneer (Daejeon, Republic of Korea). The ELISA kits for TNF-α, IL-1β, and IL-6 were obtained from BioLegend (San Diego, CA, USA). The primary antibodies were obtained from Abcam, Cell Signaling, and Santa Cruz Biotechnology. Anti-rabbit or anti-mouse IgG conjugated with horseradish peroxidase was supplied by Protein-Tech (Boston, MA, USA).

### 4.2. GP Production and Purification

Production, purification, and identification of GP from *U. pinnatifida* were conducted as previously described [26].

### 4.3. Cell Culture and Cell Viability

RAW 264.7 and peritoneal macrophage and cell culture was performed as described previously [39]. RAW 264.7 murine macrophage cells were obtained from ATCC (Manassas, VA, USA) and cultured in DMEM (Life Technologies, Grand Island, NY, USA) with 10% FBS and 1% P/S (100 units/mL penicillin and 100 μg/mL streptomycin). All animal procedures followed the Guidelines of the Committee on Laboratory Animal Ethics, Kyungpook National University (KNU 2018-0052, Daegu, Republic of Korea). The GP (5, 10, 20 µg/mL) was administrated into a RAW 264.7 cells (1 × 10^6^) (ATCC, Manassas, VA, USA), and peritoneal macrophages were cultured for 24 h at 37 °C in 5% CO_2_ in a 96-well plate. The GP’s concentration (5 to 40 µg/mL) selection was also supported by pilot experiments. Cell viability after administration of the GP was assessed using 3-(4,5-dimethylthiazol-2-yl)-2,5-diphenyl-2H-tetrazolium bromide (MTT) [39].

### 4.4. Measurement of Cytokines, NO, and PGE_2_

Predetermined doses of GP (5, 10, and 20 μg/mL) were incubated with or without LPS (0.5 μg/mL). This was incubated for 24 h within monolayers of RAW 264.7 and peritoneal cells. Following this, supernatants were carefully harvested and treated with Griess reagent at a 50:1 volume-to-volume ratio, which is crucial for quantifying NO levels, as described by Bajpai et al. (2018) [46]. IL-1β, IL-6, TNF-α, and PGE_2_ levels were quantified using ELISA kits, according to the manufacturer’s guidelines and following the methods described by Rahman et al. (2021) [28].

### 4.5. Western Blotting, RT-PCR, and the NF-κB Reporter Assay

RAW 264.7 and peritoneal cells were seeded in 12-well plates. Both cells were then maintained in DMEM before being transferred to a 37 °C incubator with CO_2_. Next, the reporter gene pRL SV40 (reporter construct) and pNF-κB-luc were combined with the transfection reagent. The mixture was then incubated at room temperature for 15 min, followed by the addition of 500 μL medium. The cells were isolated from preventive serum and subsequently exposed to different concentrations of GP (5, 10, and 20 μg/mL) for 30 min. After the addition of GP, the mixture was maintained in a CO_2_ incubator at 37 °C for 1 h. The cells were then rinsed twice with DPBS. Luciferase assays were performed using a Dual-Luciferase Reporter Assay System. ICC and confocal image analyses of NF-κB translocation were performed by Rahman et al. (2021) using immunocytochemistry [28]. The expression was assessed in all cells using various techniques, including RT-PCR, protein isolation, and Western blotting, following the methodology outlined by Rahman et al. (2021) [28].

### 4.6. Carrageenan-Induced Inflammation and Xylene-Induced Ear Edema Animal Models

Eight-week-old male mice and weighing 25–30 g were housed under controlled conditions. The temperature was maintained at 23 ± 1 °C, with a humidity level of 55 ± 5%, and a 12 h light/dark cycle. The animals received rodent food and water for one week before the trial. The Kyungpook National University IACUC approved the animal studies KNU-2023-0010 (CA-induced inflammation model) and KNU-2023.0012 (xylene-induced inflammation model), and all the criteria were followed. In vivo experiments were designed according to our previously described methodology [28], with minor modifications. We selected the dose and time based on the dose–response relationship, as it falls within the mid-range of the dose–response curve for many drugs and treatments. This makes it a reasonable starting point for observing both efficacy and potential side effects. The dose selection was also supported by pilot experiments. In each model, the mice were divided into four groups of six and administered normal drinking water: C1 and X1 (negative control group, received dH_2_O); C2 and X2 (model groups received CA and xylene, respectively); C3 and X3 (positive controls, administered indomethacin at 10 mg/kg); and C4 and X4 (sample-treated group, received sample at 10 mg/kg/day for 4 days). For the model groups (C2 and X2), 1% solution of carrageenan in saline (50 μL/mouse) was administered to the right paw, whereas 30 μL of xylene was applied to the inner surface of the right ear. The left ear was used as a control. Indomethacin was administered orally 1 h before carrageenan and xylene insults as a positive control for anti-inflammatory doses. The paw edema volume was measured using a paleothermometer (UGO BASILE Comerio, VA, Comerio, Italy), and the change in ear weight was considered to indicate ear edema. Mouse paw tissues and ears were fixed in 4% formalin, embedded in paraffin, sectioned, and stained with hematoxylin and eosin (H&E) using the standard procedure for histological analysis.

### 4.7. Statistics

Statistical significance was assessed using an unpaired *t*-test in SPSS, with results deemed significant at a two-tailed *p* value < 0.05. Differences between GP-treated and untreated or LPS-treated alone samples were further analyzed using a post hoc Tukey HSD test.

## 5. Conclusions

This study highlights the potential of GP from *Undaria pinnatifida* as a natural anti-inflammatory agent. GP effectively reduces key inflammatory markers, including iNOS and COX-2, and inhibits pro-inflammatory cytokine production by suppressing NF-κB and MAPK signaling pathways. Its efficacy in reducing inflammation and edema in animal models underscores the therapeutic promise of GP. These findings pave the way for developing GP-based treatments for inflammatory diseases, presenting a viable alternative to conventional NSAIDs with fewer adverse effects. Future studies should focus on the detailed structural and functional analysis of GP to fully elucidate its pharmacological potential.

## Data Availability

The data presented in this study are available on request from the corresponding author.
